# Editorial: Immunotherapies Towards HIV Cure

**DOI:** 10.3389/fcimb.2021.655363

**Published:** 2021-05-11

**Authors:** Maria Salgado, Alberto Bosque, Carolina Garrido

**Affiliations:** ^1^ IrsiCaixa AIDS Research Institute, Barcelona, Spain; ^2^ Department of Microbiology, Immunology and Tropical Medicine, George Washington University, Washington, DC, United States; ^3^ Department of Medicine, University of North Carolina at Chapel Hill, Chapel Hill, NC, United States

**Keywords:** HIV, Immunotheraphy, reservoir, shock and kill, CD8+T cells, NK cells, HIV cure, HIV eradication

Despite the clinical success of antiretroviral therapy (ART), there is still no cure for HIV infection. If treatment is interrupted, viremia almost inevitably rebounds, and without renewed therapy, it can lead to opportunistic infections, AIDS and death. Thus, research towards an ART free remission of HIV infection, or cure, is a high priority.

The main obstacle to achieve a cure for HIV infection rests in the early establishment of a pool of long-lived cells harboring an intact HIV genome that has entered latency. Significant advancements in the understanding of persistent infection have emerged over the last years, shedding light into possible mechanisms to tackle it. Among these mechanisms, strategies to enhance the immune system to allow the clearance of latently HIV infected cells is of special interest. Some of the ultimate immunotherapy approaches that have obtained relevant results are published in this special number.

One of the first attempts to eliminate the HIV reservoir consisted on the reactivation of the latently infected cells using epigenetic modulators (Spivak and Planelles, 2016). Although this strategy proved effective in awakening the dormant cells, it also resulted unsuccessful in reducing the size of the reservoir. Thus, it was obvious that additional strategies to clear the reactivated reservoir needed to be implemented, as well as finding more potent latency reversal agents (LRAs). One ideal solution to the problem would be the development of a drug that simultaneously reactivate the latent reservoir and stimulate the immune system. Toll-like receptor (TLR) agonists could fit this description, as reviewed by Martinsen et al. Another option would be a combined regimen including an LRA with a immune modulator, like Mothe et al. and Rosas-Umbert et al. evaluated in a clinical trial, assessing the combination of the LRA Romidepsin with a therapeutic vaccine. They observed reactivation of the reservoir to some extent and enhanced activation of HIV specific T cells, which led to a marginal reduction in the size of the reservoir. However, in the mentioned studies, immune assessment was focused on CD8 T cells, while increasing body of evidence suggest that other immune subsets are likely to contribute to viral clearance in the context of HIV eradication, such as NK cells, as demonstrated by May et al. in their *in vitro* experiments and reviewed by Mann et al. in a broader innate immune system perspective. Another alternative to enhance immune clearance of HIV is to use adoptive cell immunotherapies. In particular, the use of antigen specific T cells for other viral infections post hematopoietic stem cell transplantation (Lee et al.). Chimeric antigen receptors (CARs) are another alternative being tested to directly kill infected cells. This approach was first explored in the oncology field and now is advancing into the HIV arena. Hajduczki et al. demonstrate that a trispecific CD4-based CAR expressing a carbohydrate recognition domain of human mannose-binding lectin (MBL) and a third targeting moiety against a conserved Env determinant, exhibited high anti-HIV potency as well as undetectable HIV entry receptor activity. Importantly, properly detect and monitor immune responses in these trials is essential to evaluate the duration of the adopted immune response. The use of a newly developed assay named ViraFEST by Chan et al. could provide a platform to evaluate memory CD8T cells responses. Excitingly, improvement of LRA activity and reservoir clearance could be facilitated by the field of immunoengineering, as reviewed by Bowen et al.

The potential of antibodies in HIV clearance strategies is under testing and some studies have shown promising results. A step further in this concept is the development of engineered molecules to mediate viral recognition for clearance. Dual-Affinity Re-Targeting (*DART*) molecules were initially designed with an arm to engage HIV infected cells and an arm to engage T cells from HIV infected adults (Sung et al., 2015). Pollara et al. evaluate these molecules with T cells from cord blood, to investigate potential pediatric use, and furthermore, a new version of these molecules targeting CD16 expressing effector cells are examined, proving to eliminate HIV-infected cells specially after IL-15 priming. However, delivery of antibodies and other immune products remains challenging, specially thinking about long-term supply. Martinez-Navio et al. showed how adeno-associated virus (AAV) can be used as a vector to deliver anti-SIV antibodies allowing IgG detection for over 6 years, showing promise not only for antibody delivery but for other immunomodulators. Moreover, the use of antibodies for cure approaches is not restricted to HIV antibodies. For example, α4β7 ntegrin promotes homing of activated T cells to intestinal sites where they become preferentially HIV infected. Their targeting using anti-α4β7 monoclonal antibody has been proposed as means to reduce viral infection and gut inflammation, as evaluated in Rhesus macaques by Pino et al.

It is crucial to define a reliable measure of the size of the reservoir to allow quantification of the clearance strategy used. Several measures have been studied over the years, all of them presenting with limitations, and thus further development is warranted. Stuelke et al. propose a novel assay in which is combined the principle of the quantitative viral outgrowth assay (QVOA) with ultrasensitive detection, allowing the assay to be performed with minimal sample and in a reduced time than the classic assay.

In summary, immunotherapy approaches represent an area of active research towards an ART free remission or cure of HIV. The next five years will be critical to define which immunotherapy approaches have a substantial effect in the latent reservoir. As we search for an HIV cure or a long-term remission using immunotherapy approaches, we will need to ensure that implementation of such strategies will be equitable among PLWH not only in developed countries but also in developing ones. Efforts need to be done to include diversity when clinically evaluating these approaches and to reduce the costs associated with some of these strategies so it does not halt implementation in low- and middle-income countries. Ending the HIV epidemic needs to be a global endeavor and including cure strategies on the tool box against this common enemy is a scientific priority.

## Author Contributions

All authors contributed to the article and approved the submitted version.

## Conflict of Interest

The authors declare that the research was conducted in the absence of any commercial or financial relationships that could be construed as a potential conflict of interest.
